# Hybrid nanofluid flow towards a stagnation point on a stretching/shrinking cylinder

**DOI:** 10.1038/s41598-020-66126-2

**Published:** 2020-06-09

**Authors:** Iskandar Waini, Anuar Ishak, Ioan Pop

**Affiliations:** 1 Fakulti Teknologi Kejuruteraan Mekanikal dan Pembuatan, Universiti Teknikal Malaysia Melaka, Hang Tuah Jaya, 76100 Durian Tunggal, Melaka, Malaysia; 2 Department of Mathematical Sciences, Faculty of Science and Technology, Universiti Kebangsaan Malaysia, 43600 UKM, Bangi, Selangor Malaysia; 3 Department of Mathematics, Babeş-Bolyai University, 400084 Cluj-Napoca, Romania

**Keywords:** Applied mathematics, Fluid dynamics

## Abstract

This paper examines the stagnation point flow towards a stretching/shrinking cylinder in a hybrid nanofluid. Here, copper (Cu) and alumina (Al_2_O_3_) are considered as the hybrid nanoparticles while water as the base fluid. The governing equations are reduced to the similarity equations using a similarity transformation. The resulting equations are solved numerically using the boundary value problem solver, bvp4c, available in the Matlab software. It is found that the heat transfer rate is greater for the hybrid nanofluid compared to the regular nanofluid as well as the regular fluid. Besides, the non-uniqueness of the solutions is observed for certain physical parameters. It is also noticed that the bifurcation of the solutions occurs in the shrinking regions. In addition, the heat transfer rate and the skin friction coefficients increase in the presence of nanoparticles and for larger Reynolds number. It is found that between the two solutions, only one of them is stable as time evolves.

## Introduction

Boundary layer flow caused by the stretching/shrinking surface has been practiced in industrial engineering and manufacturing processes. To name a few, the polymer or metal extrusions, wire drawing, and continuous glass casting are such processes that involved these kinds of surfaces^1,2^. Historically, it seems that Crane^3^ is the first researcher to examine the flow over a linearly stretching surface. Instead of a stretching surface, the problems of a shrinking surface have become the topic of interest in the last few years. The flow caused by the shrinking surface is fundamentally a reverse flow as pondered by Goldstein^4^. This type of flow is different from the stretching surface owing to the existence of the vorticity inside the boundary layer, which discovered by Wang^5^. Thus, some other outside force is required to overcome this situation and only then the steady flow is possible. In the work of Miklavčič and Wang^6^, they suggested that the flow can be preserved by the application of suction on the surface. Besides, the existence of the stagnation flow velocity can confine the vorticity to maintain the flow, thus the application of suction on the shrinking sheet is not necessary as discussed by Wang^7^. The stagnation point flow describes the fluid movement close to the stagnation region of a solid surface.

The problem of stagnation point flow towards a cylinder has been also considered by many researchers. For example, Wang^8^ considered the stagnation flow towards a circular cylinder. Then, Wang^9^ extended this problem by considering partial slip condition on the cylinder surface. After that, Gorla^10^ examined a similar problem of Wang^8^ but with the effect of Prandtl numbers on the heat transfer. Then, Cunning *et al*.^11^ investigated the transpiration and rotation effects on the stagnation flow towards a circular cylinder. However, there is a limited reference on the flow over a shrinking cylinder in the literature. In this respect, Lok and Pop^12^ investigated this problem by considering the stagnation point flow and suction effects. Merkin *et al*.^13^ studied the stagnation-point flow and heat transfer over an exponentially stretching/shrinking cylinder. Further, Zaimi *et al*.^14^ examined the effect of suction on the unsteady flow over a shrinking cylinder and found the dual solutions exist for a certain range of suction and unsteadiness parameters. Besides, Soomro *et al*.^15^ examined the nanofluid flow along a permeable shrinking vertical cylinder with slip effects. Other than that, some other useful studies to simulate the flow over a cylinder under different conditions can be found in the literature^16–21^.

Nowadays, the problem of heat transfer enhancement in industrial processes has become the main topic of interest to the researchers. Previously, the fluids like water, oil, and ethylene glycol were regularly considered as a cooling liquid in those processes, but their heat transfer rates are low. In 1995, Choi and Eastman^22^ introduced the fluid called ‘nanofluid’ to replace the use of regular fluids in the industrial processes. Nanofluids are engineered by dispersing one type of nanoparticle in the aforementioned fluids to enhance their thermal conductivity. Khanafer *et al*.^23^ and Oztop and Abu-Nada^24^ have utilized nanofluids to study the heat transfer enhancement in a rectangular enclosure. However, the researchers found that the thermal properties of the nanofluid could be improved with the addition of more than a single nanoparticle in the base fluid and named it ‘hybrid nanofluid’. The experimental studies that considered the hybrid nano-composite particles have been conducted by several researchers, for example, Turcu *et al*.^25^ and Jana *et al*.^26^. Hybrid nanofluid is an advanced fluid that incorporates more than one nanoparticle which has the capacity of raising the heat transfer rate because of the synergistic effects^27^.

Furthermore, the studies of hybrid nanofluid were extended to the boundary layer flow problem. For instance, Devi and Devi^28,29^ started to examine the advantages of utilizing hybrid nanofluid over a stretching surface. They found that the heat transfer rate was intensified in the presence of the hybrid nanoparticles. In their studies, the new thermophysical model was introduced and validated with the experimental data of Suresh *et al*.^30^. Furthermore, Waini *et al*.^31^ examined the stability of the multiple solutions of the flow over a stretching/shrinking surface in a fluid containing hybrid nanoparticles. They discovered that only one of the solutions is stable and thus physically reliable as time evolves. Besides, Waini *et al*.^32–37^ in a series of papers have extended the problem to different surfaces. Moreover, the effects of MHD and viscous dissipation have been studied by Lund *et al*.^38^, considering Cu–Fe_3_O_4_/H_2_O hybrid nanofluid in a porous medium. Additionally, the problem of hybrid nanofluid flow with the effect of different physical parameters was also considered by several authors^39–45^.

Thus, the objective of this paper is to examine the hybrid nanofluid flow towards a stagnation point on a stretching/shrinking cylinder. Here, copper (Cu) and alumina (Al_2_O_3_) are considered as the hybrid nanoparticles, while water as the base fluid.

## Mathematical Model

**Figure 1:**
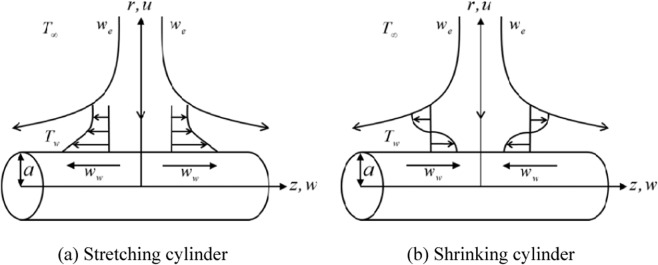
Physical configuration.

Consider a hybrid nanofluid flow towards a stagnation point on a stretching/shrinking cylinder with radius *a* as illustrated in Fig. 1. Here, (*z*,*r*) is the cylindrical polar coordinates which assigned in the axial and radial directions, respectively. The flow is assumed to be symmetric about the *z* = 0 plane and also axisymmetric about the *z*-axis, with the stagnation line is at *z* = 0 and *r* = *a*. The surface velocity of the cylinder is given as *w*_*w*_(*z*) = 2*bz* where the static cylinder is denoted by *b* = 0, whereas the cylinder is stretched or shrunk when *b* > 0 or *b* < 0, respectively. Meanwhile, the free stream velocity is taken as *w*_*e*_(*z*) = 2*cz* where *c* > 0. Moreover, the surface temperature *T*_*w*_ and the ambient temperature *T*_∞_ are constant, where *T*_*w*_ > *T*_∞_. Also, it is assumed that the shape of the nanoparticle is spherical and its size is uniform, while the agglomeration is disregarded since the hybrid nanofluid is formed as a stable composite. Therefore, the equations that govern the hybrid nanofluid flow are (see Wang^8^, Lok and Pop^12^):

1 $$\frac{\partial (rw)}{\partial z}+\frac{\partial (ru)}{\partial r}=0$$

2 $$w\frac{\partial w}{\partial z}+u\frac{\partial w}{\partial r}={w}_{e}\frac{d{w}_{e}}{dz}+\frac{{\mu }_{hnf}}{{\rho }_{hnf}}\left(\frac{{\partial }^{2}w}{\partial {r}^{2}}+\frac{1}{r}\frac{\partial w}{\partial r}\right)$$

3 $$w\frac{\partial T}{\partial z}+u\frac{\partial T}{\partial r}=\frac{{k}_{hnf}}{{(\rho {C}_{p})}_{hnf}}\left(\frac{{\partial }^{2}T}{\partial {r}^{2}}+\frac{1}{r}\frac{\partial T}{\partial r}\right)\,$$

subject to:

$$u=0,w={w}_{w},\,T={T}_{w}\,{\rm{at}}\,r=a$$

4 $$w\to {w}_{e},T\to {T}_{\infty }\,{\rm{as}}\,r\to \infty $$

where *w* and *u* represent the velocity components along the *z*- and *r*- axes, and *T* represents the temperature of the hybrid nanofluid. Further, the thermophysical properties of the hybrid nanofluid are defined in Table 1 ^24,28,31^. Besides, the physical properties of Al_2_O_3_, Cu, and water are provided in Table 2 ^24,31^. Here, Al_2_O_3_ and Cu volume fractions are given by *φ*_1_ and *φ*_2_ and the subscripts *n*1 and *n*2 correspond to their solid components, respectively. Meanwhile, the fluid, nanofluid, and the hybrid nanofluid are designated by the subscripts *f*, *nf*, and *hnf*, respectively.

**Table 1:** Thermophysical properties of nanofluid and hybrid nanofluid^24,28,31^.

Thermophysical Properties	Nanofluid	Hybrid nanofluid
Density	${\rho }_{nf}=(1-{\varphi }_{1}){\rho }_{f}+{\varphi }_{1}{\rho }_{n1}$	${\rho }_{hnf}=(1-{\varphi }_{2})[(1-{\varphi }_{1}){\rho }_{f}+{\varphi }_{1}{\rho }_{n1}]+{\varphi }_{2}{\rho }_{n2}$
Heat capacity	${(\rho {C}_{p})}_{nf}=(1-{\varphi }_{1}){(\rho {C}_{p})}_{f}+{\varphi }_{1}{(\rho {C}_{p})}_{n1}$	${(\rho {C}_{p})}_{hnf}=(1-{\varphi }_{2})[(1-{\varphi }_{1}){(\rho {C}_{p})}_{f}+\,{\varphi }_{1}{(\rho {C}_{p})}_{n1}]+{\varphi }_{2}{(\rho {C}_{p})}_{n2}$
Dynamic viscosity	${\mu }_{nf}=\frac{{\mu }_{f}}{{(1-{\varphi }_{1})}^{2.5}}$	${\mu }_{hnf}=\frac{{\mu }_{f}}{{(1-{\varphi }_{1})}^{2.5}\,{(1-{\varphi }_{2})}^{2.5}}$
Thermal conductivity	$\frac{{k}_{nf}}{{k}_{f}}=\frac{{k}_{n1}+2{k}_{f}-2{\varphi }_{1}({k}_{f}-{k}_{n1})}{{k}_{n1}+2{k}_{f}+{\varphi }_{1}({k}_{f}-{k}_{n1})}$	$\frac{{k}_{hnf}}{{k}_{nf}}=\frac{{k}_{n2}+2{k}_{nf}-2{\varphi }_{2}({k}_{nf}-{k}_{n2})}{{k}_{n2}+2{k}_{nf}+{\varphi }_{2}({k}_{nf}-{k}_{n2})}$ where $\frac{{k}_{nf}}{{k}_{f}}=\frac{{k}_{n1}+2{k}_{f}-2{\varphi }_{1}({k}_{f}-{k}_{n1})}{{k}_{n1}+2{k}_{f}+{\varphi }_{1}({k}_{f}-{k}_{n1})}$

**Table 2:** Thermophysical properties of nanoparticles and water^24,31^.

Thermophysical Properties	Al_2_O_3_	Cu	water
*ρ*(*kg*/*m*^3^)	3970	8933	997.1
*C*_*p*_(*J*/*kgK*)	765	385	4179
*k*(*W*/*mK*)	40	400	0.613
Prandtl number, Pr			6.2

An appropriate transformation is introduced as follows (see Wang^8^, Lok and Pop^12^):

5 $$u=-caf(\eta )/\sqrt{\eta },w=2czf{\prime} (\eta ),\theta (\eta )=\frac{T-{T}_{\infty }}{{T}_{w}-{T}_{\infty }},\,\eta ={\left(\frac{r}{a}\right)}^{2}$$

Employing these definitions, Eq. (1) is identically fulfilled. Then, the following similarity equations are obtained:

6 $$\frac{{\mu }_{hnf}/{\mu }_{f}}{{\rho }_{hnf}/{\rho }_{f}}(\eta f{\prime\prime} +f{\prime\prime} )+Re(ff{\prime\prime} -{f}^{{\prime} 2}+1)=0$$

7 $$\frac{1}{{\rm{\Pr }}}\frac{{k}_{hnf}/{k}_{f}}{{(\rho {C}_{p})}_{hnf}/{(\rho {C}_{p})}_{f}}(\eta \theta {\prime\prime} +\theta {\prime} )+Ref\theta {\prime} =0$$

subject to:

$$f(1)=0,f{\prime} (1)=\varepsilon ,\theta (1)=1$$

8 $$f{\prime} (\infty )=1,\theta (\infty )=0$$

where (′) represents the differentiation with respect to *η*, *Re* = *ca*^2^/2*ν*_*f*_ represents the Reynolds number, and $\Pr =\,{\mu }_{f}{({C}_{p})}_{f}/{k}_{f}$ represents the Prandtl number. Besides, the stretching/shrinking parameter symbolized by *ε* = *b*/*c* with *ε* > 0 and *ε* < 0 are for stretching and shrinking cylinder, respectively, while the static cylinder is denoted by *ε* = 0.

The skin friction coefficient *C*_*f*_ and the Nusselt number *Nu* are defined as:

9 $${C}_{f}=\frac{2{\mu }_{hnf}}{{\rho }_{f}\,{w}_{e}^{2}}{\left(\frac{\partial w}{\partial r}\right)}_{r=a},\,Nu=-\,\frac{a\,{k}_{hnf}}{{k}_{f}({T}_{w}-\,{T}_{\infty })}{\left(\frac{\partial T}{\partial r}\right)}_{r=a}$$

Inserting (5) into (9), one obtains

10 $$\left(\frac{Rez}{a}\right){C}_{f}=\frac{{\mu }_{hnf}}{{\mu }_{f}}f{\prime\prime} (1),\,Nu=-2\frac{{k}_{hnf}}{{k}_{f}}\theta {\prime} (1)$$

## Stability Analysis

The existence of the non-uniqueness solutions of Eqs. (6) to (8) is observed for a certain range of the physical parameters. A temporal stability analysis is therefore needed to determine which solution is stable and thus physically reliable as time evolves. This technique was initiated by Merkin^46^ in 1986. A dimensionless time variable *τ* was introduced by Weidman *et al*.^47^ to further study the stability of the solutions in the long run. They concluded that the upper branch (first) solutions are stable, while the lower branch (second) solutions are unstable. The new variables based on Eq. (5) are given as follows:

11 $$u=-\,caf(\eta ,\tau )/\sqrt{\eta },w=2cz\frac{\partial f}{\partial \eta }(\eta ,\tau ),\theta (\eta ,\tau )=\frac{T-\,{T}_{\infty }}{{T}_{w}-\,{T}_{\infty }},\eta ={\left(\frac{r}{a}\right)}^{2},\tau =2ct$$

To study the stability of the solutions of Eqs. (1) to (3), the unsteady form of these equations are considered. Using (11) and following the same procedure as previous section, the equations transformed to:

12 $$\frac{{\mu }_{hnf}/{\mu }_{f}}{{\rho }_{hnf}/{\rho }_{f}}\left(\eta \frac{{\partial }^{3}f}{\partial {\eta }^{3}}+\frac{{\partial }^{2}f}{\partial {\eta }^{2}}\right)+Re\left(f\frac{{\partial }^{2}f}{\partial {\eta }^{2}}-{\left(\frac{\partial f}{\partial \eta }\right)}^{2}+1-\frac{{\partial }^{2}f}{\partial \eta \partial \tau }\right)=0$$

13 $$\frac{1}{{\rm{\Pr }}}\frac{{k}_{hnf}/{k}_{f}}{{(\rho {C}_{p})}_{hnf}/{(\rho {C}_{p})}_{f}}\left(\eta \frac{{\partial }^{2}\theta }{\partial {\eta }^{2}}+\frac{\partial \theta }{\partial \eta }\right)+Re\left(f\frac{\partial \theta }{\partial \eta }-\frac{\partial \theta }{\partial \tau }\right)=0$$

subject to:

$$f(1,\tau )=0,\frac{\partial f}{\partial \eta }(1,\tau )=\varepsilon ,\theta (1,\tau )=1$$

14 $$\frac{\partial f}{\partial \eta }(\infty ,\tau )=1,\theta (\infty ,\tau )=0$$

To examine the stability behaviour, the disturbance is imposed to the steady solution *f* = *f*_0_(*η*) and *θ* = *θ*_0_(*η*) of Eqs. (6) to (8) by using the following relations (see Weidman *et al*.^47^):

15 $$f(\eta ,\tau )={f}_{0}(\eta )+{e}^{-\gamma \tau }F(\eta ),\theta (\eta ,\tau )={\theta }_{0}(\eta )+{e}^{-\gamma \tau }G(\eta )$$

where *γ* indicates the unknown eigenvalue that determines the stability of the solutions, whereas *F*(*η*) and *G*(*η*) are small compared to *f*_0_(*η*) and *θ*_0_(*η*). The disturbance is taken exponentially as it demonstrates the rapid decline or development of the disturbance. By employing Eq. (15), Eqs. (12) and (13) become

16 $$\frac{{\mu }_{hnf}/{\mu }_{f}}{{\rho }_{hnf}/{\rho }_{f}}(\eta {F}^{\text{'}\text{'}\text{'}}+{F}^{\text{'}\text{'}})+Re({f}_{0}F{\prime\prime} +{f{\prime\prime} }_{0}F-2{f{\prime} }_{0}F{\prime} +\gamma F{\prime} )=0$$

17 $$\frac{1}{{\rm{\Pr }}}\frac{{k}_{hnf}/{k}_{f}}{{(\rho {C}_{p})}_{hnf}/{(\rho {C}_{p})}_{f}}(\eta G{\prime\prime} +G{\prime} )+Re({f}_{0}G{\prime} +{\theta {\prime} }_{0}F+\gamma G)=0$$

subject to:

$$F(1)=0,F{\prime} (1)=0,G(1)=0$$

18 $$F{\prime} (\infty )=0,G(\infty )=0$$

Without loss of generality, the values of *γ* from Eqs. (16) to (18) are obtained for the case of *F*″(1) = 1 as proposed by Harris *et al*.^48^.

## Numerical Method

The boundary value problem solver, bvp4c, available in the Matlab software is utilized for solving Eqs. (6) to (8), numerically. As described in Shampine *et al*.^49^, the aforementioned solver is a finite difference method that employs the 3-stage Lobatto IIIa formula. The selection of the initial guess and the boundary layer thickness *η*_∞_ depend on the parameter values applied to obtain the required solutions. Moreover, several researchers^50–55^ were also employing this solver for solving the boundary layer flow problems. First, Eqs. (6) and (7) are reduced to a system of ordinary differential equations of the first order. Equation (6) is written as:

$$f=y(1)$$

19a $$f{\prime} =y{\prime} (1)=y(2)$$

19b $$f{\prime\prime} =y{\prime} (2)=y(3)$$

19c $$f\prime\prime\prime =y{\prime} (3)=-\frac{1}{\eta }\left\{\frac{{\rho }_{hnf}/{\rho }_{f}}{{\mu }_{hnf}/{\mu }_{f}}Re(y(1)y(3)-y{(2)}^{2}+1)+y(3)\right\}$$

while Eq. (7) becomes

$$\theta =y(4)$$

20a $$\theta {\prime} =y{\prime} (4)=y(5)$$

20b $$\theta {\prime\prime} =y{\prime} (5)=-\frac{1}{\eta }\left\{{\rm{\Pr }}\frac{{(\rho {C}_{p})}_{hnf}/{(\rho {C}_{p})}_{f}}{{k}_{hnf}/{k}_{f}\,}Rey(1)y(5)+y(5)\right\}$$

with the boundary conditions:

$$ya(1)=0,ya(2)=\varepsilon ,ya(4)=1$$

21 $$yb(2)=1,\,yb(4)=0$$

Then, Eqs. (19) to (21) are coded in Matlab software to obtain the required solutions.

## Results and Discussion

In the present study, the volume fractions of Cu are varied from 0 to 0.04 (0 ≤ *φ*_2_ ≤ 0.04), while the volume fraction of Al_2_O_3_ is kept fixed at *φ*_1_ = 0.04 and water as the base fluid. Table 3 provides the numerical values of *f*″(1) and −2*θ*'(1) under different values of *Re* for regular fluid (*φ*_1_ = *φ*_2_ = 0) when *ε* = 0 and Pr = 6.2. In the present study, *η*_∞_ = 35 is sufficient for the velocity and temperature profiles to reach the far-field boundary conditions asymptotically, which supports the validity of the numerical results. The increase of *f*″(1) and −2*θ*'(1) are observed as *Re* increases. Also, the comparison values of *f*″(1) with those of Wang^8,9^ and Lok and Pop^12^ are shown in the same table. It is observed that the comparison is in excellent agreement with the mentioned literature which supports the validity of the present numerical results. Additionally, Table 4 shows the effects of *φ*_2_, *Re*, and *ε* on the skin friction coefficient (*Rez*/*a*)*C*_*f*_ and the Nusselt number *Nu* for Cu/water (*φ*_1_ = 0) and Al_2_O_3_-Cu/water (*φ*_1_ = 0.04) when Pr = 6.2. It is observed that the values of (*Rez*/*a*)*C*_*f*_ and *Nu* increase with the increasing values of *φ*_2_ and *Re*. Besides, the values of (*Rez*/*a*)*C*_*f*_ decrease, whereas *Nu* increases with increasing values of *ε*. Also, the heat transfer rate for Al_2_O_3_-Cu/water hybrid nanofluid is intensified if compared to Cu/water nanofluid.

**Table 3:** Values of f″(1) and −2θ'(1) under different values of Re for regular fluid (*φ*_1_ = *φ*_2_ = 0) when  *ε* = 0 and Pr=6.2.

Re	f″(1)				*f*) -2θ'(1)
	Wang^8^	Wang^9^	Lok and Pop^12^	Present results	Present results
0.2	0.78605	0.78604	0.786042	0.786042	1.508635
1	1.484185	1.48418	1.484183	1.484183	2.793424
10	4.16292	4.16292	4.162920	4.162920	7.701472

**Table 4:** Values of (*Rez*/*a*)*C*_*f*_  and Nu for Cu/water (φ_1_ = 0) and Al_2_O_3_-Cu/water (*φ*_1_ = 0.04) under different values of physical parameters. when Pr = 6.2.

*φ*_2_	Re	*ε*	Cu/water (φ_1_ =0)		Al_2_O_3_-Cu/water (*φ*_1_ = 0.04)	
			(*Rez*/*a*)*C*_*f*_	*Nu*	(*Rez*/*a*)*C*_*f*_	Nu
0	0.2	0	0.786042	1.508635	0.873892	1.632938
0.02			0.856892	1.584409	0.946858	1.712793
0.04			0.928449	1.660081	1.021036	1.792922
0.04	0.5		1.326543	2.330191	1.457949	2.509315
	1		1.769560	3.072779	1.944092	3.302625
	2		2.391980	4.113770	2.627041	4.414274
	1	−0.5	2.266010	2.074241	2.490374	2.279514
		0.5	0.994933	3.924611	1.092842	4.177081
		1.5	−1.181197	5.325017	−1.297131	5.621329

**Figure 2:**
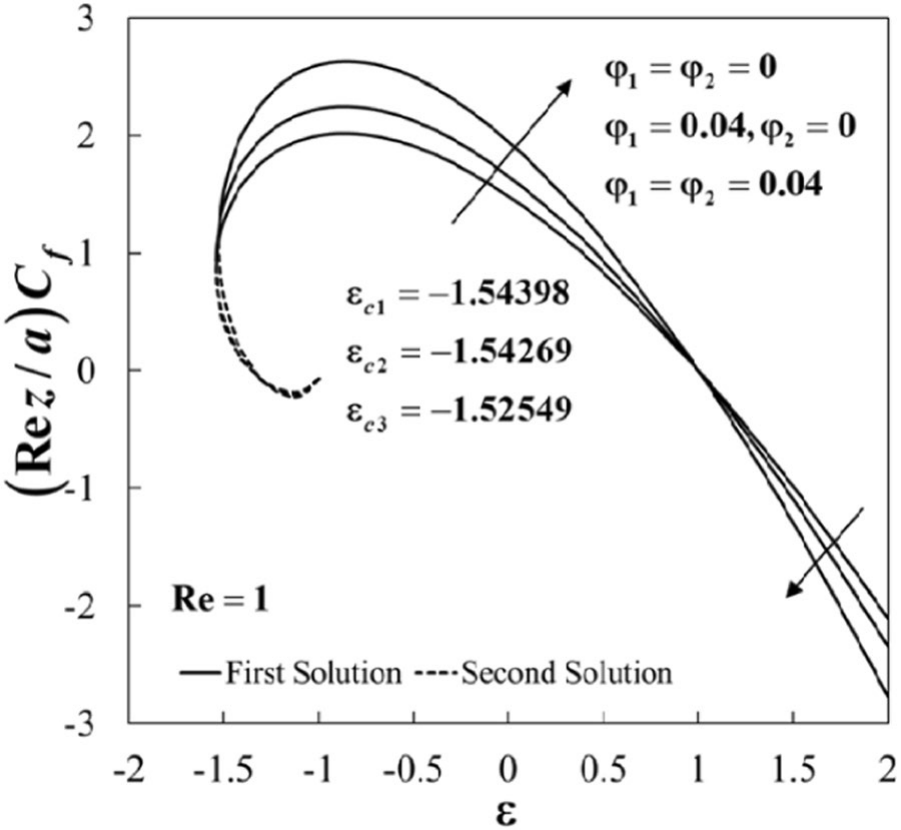
Plot of (*Re* *z*/*a*)*C*_*f*_ against *ε* for different values of *φ*_1_ and *φ*_2_.

**Figure 3:**
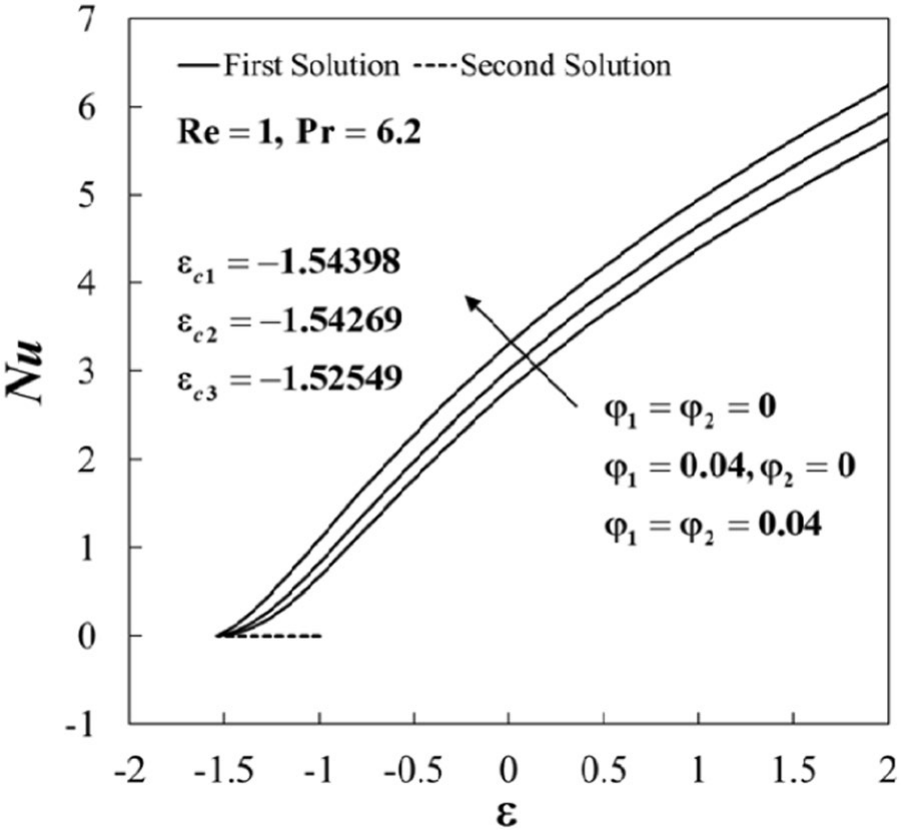
Plot of *Nu* against *ε* for different values of *φ*_1_ and *φ*_2_.

**Figure 4:**
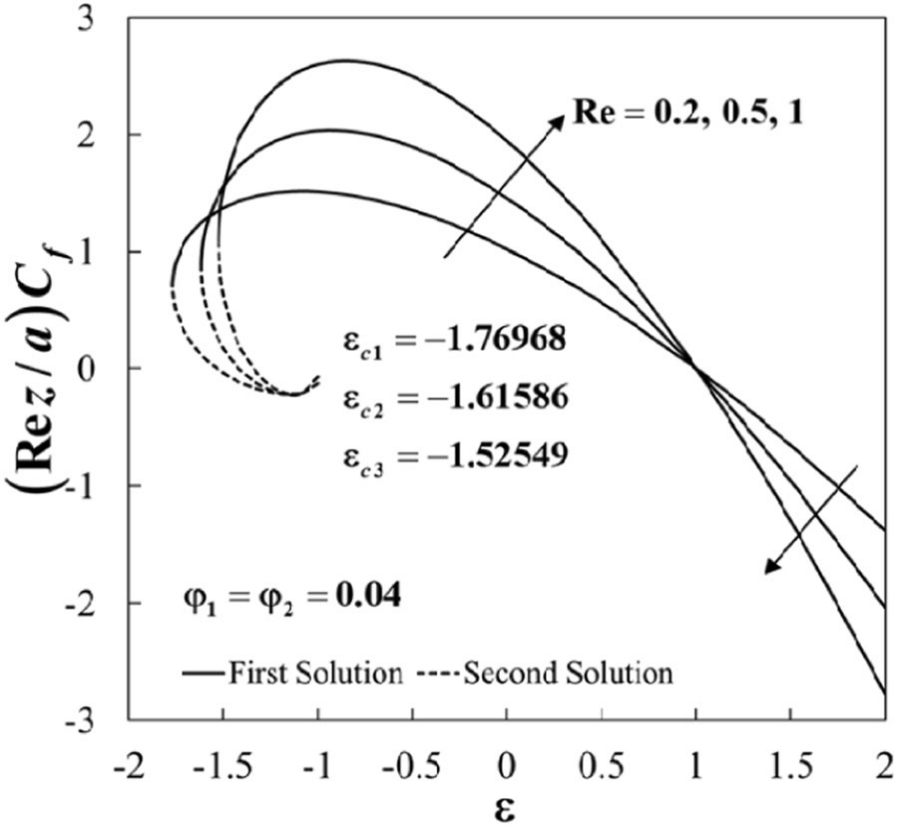
Plot of (*Rez*/*a*)*C*_*f*_ against *ε* for different values of *Re*.

**Figure 5:**
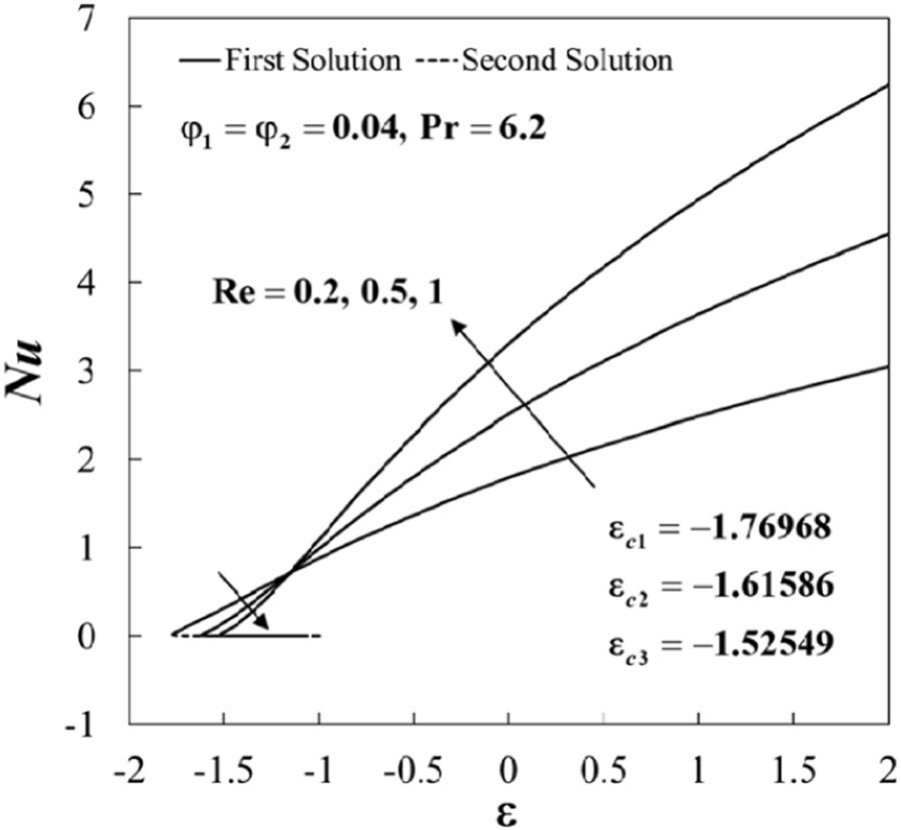
Plot of *Nu* against *ε* for different values of *Re*.

The non-uniqueness of the solutions of Eqs. (6) to (8) is observed for some values of *ε* as can be seen in Figs. 2–5. For example, the variations of (*Rez*/*a*)*C*_*f*_ and *Nu* against *ε* for several values of *φ*_1_ and *φ*_2_ when Re = 1 and $\Pr =6.2$ are displayed in Figs. 2 and 3. It is noticed that dual solutions are possible for *ε*_*c*_ < *ε* < −1, and the solution is unique for *ε* ≥ −1. Besides, both branch solutions (first and second) merge up to certain critical values of *ε*, say *ε*_*c*_. Here, *ε*_*c*1_ = −1.54398, *ε*_*c*2_ = −1.54269, and *ε*_*c*3_ = −1.52549 are the critical values for the case of regular fluid (*φ*_1_ = *φ*_2_ = 0), Al_2_O_3_/water nanofluid (*φ*_1_ = 0.04, *φ*_2_ = 0), and Al_2_O_3_-Cu/water hybrid nanofluid (*φ*_1_ = *φ*_2_ = 0.04), respectively. In addition, the Nusselt number *Nu* enhances for both stretching and shrinking cases in the presence of nanoparticles. However, the skin friction (*Rez*/*a*)*C*_*f*_ increases for fixed values of *ε* started from *ε* < 1, but these values decrease for *ε* > 1 and zero skin friction is observed for *ε* = 1. Comparing the three types of fluid, it is found that these physical quantities are intensified for hybrid nanofluid compared to the others. The observation is consistent with the fact that the added hybrid nanoparticles have the capacity of raising the heat transfer rate because of the synergistic effects as discussed by Sarkar *et al*.^27^.

Figures 4 and 5 show the variations of the skin friction coefficient (*Rez*/*a*)*C*_*f*_ and the Nusselt number *Nu* against *ε* for several values of *Re* when *φ*_1_ = *φ*_2_ = 0.04 and $\Pr =6.2$. The effect of increasing *Re* has a similar trend if compared to the effect of nanoparticles on the skin friction (*Rez*/*a*)*C*_*f*_. Physically, Reynolds number *Re* indicates the relative significance of the inertia effect compared to the viscous effect. As expected, the skin friction coefficients (the surface shear-stress) increases for increasing values of the Reynolds number *Re*. The upsurge of *Re* has a tendency to enhance the Nusselt number *Nu* for *ε* > −1.2, and it decreases for *ε* < −1.2, where the heat transfer occurs almost at the same rate as *ε* = −1.2. Also, it is noticed that the increment is more dominant for the stretching case (*ε* > 0). Besides, the range of ε for which the solution is in existence decreases as *Re* increases. As shown in Figs. 4 and 5, the minimum values of ε for the solution to exist are *ε*_*c*1_ = −1.76968, *ε*_*c*2_ = −1.61586, and *ε*_*c*3_ = −1.52549 for $\mathrm{Re}=0.2,0.5,1$, respectively.

**Figure 6:**
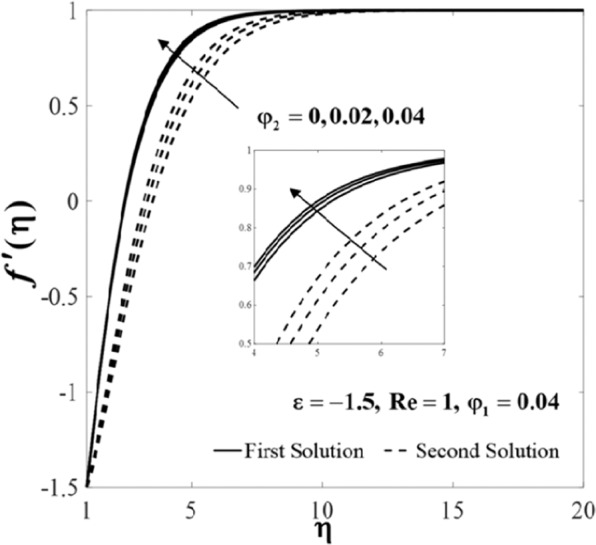
Plot of *f*′(*η*) for different values of *φ*_2_.

**Figure 7:**
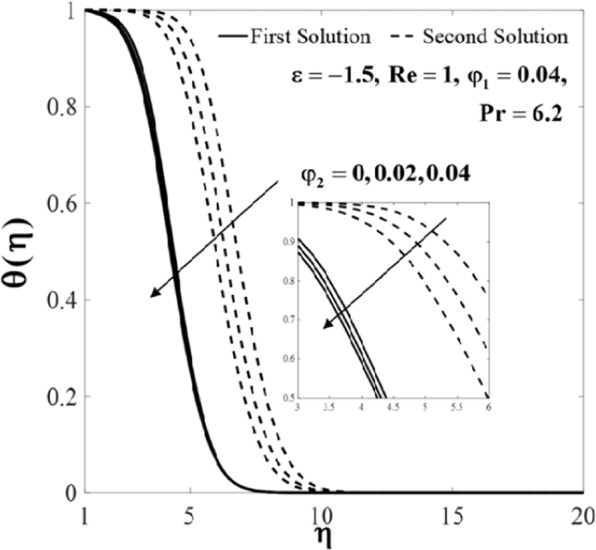
Plot of *θ*(*η*)_*f*_ for different values of *φ*_2_.

**Figure 8:**
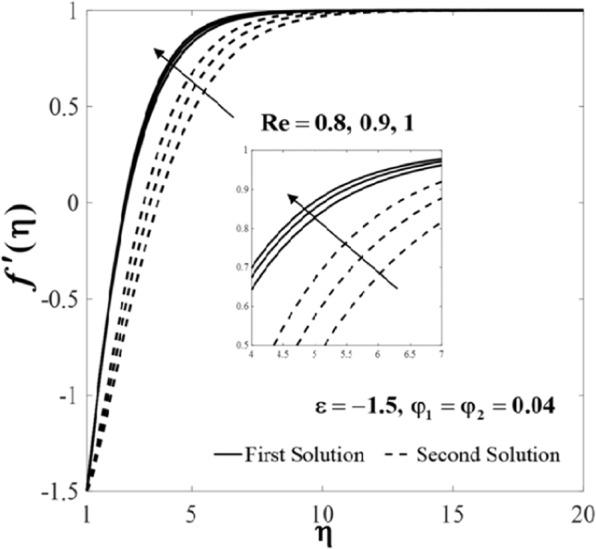
Plot of *f*′(*η*) for different values of Re.

**Figure 9:**
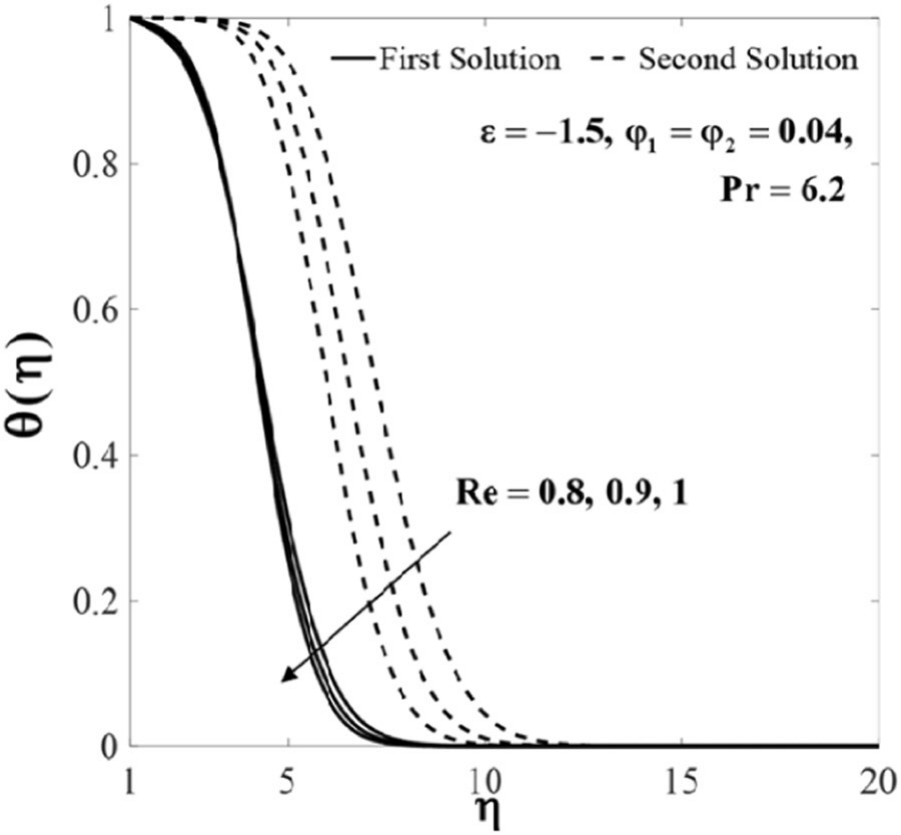
Plot of *θ*(*η*) for different values of Re.

**Figure 10:**
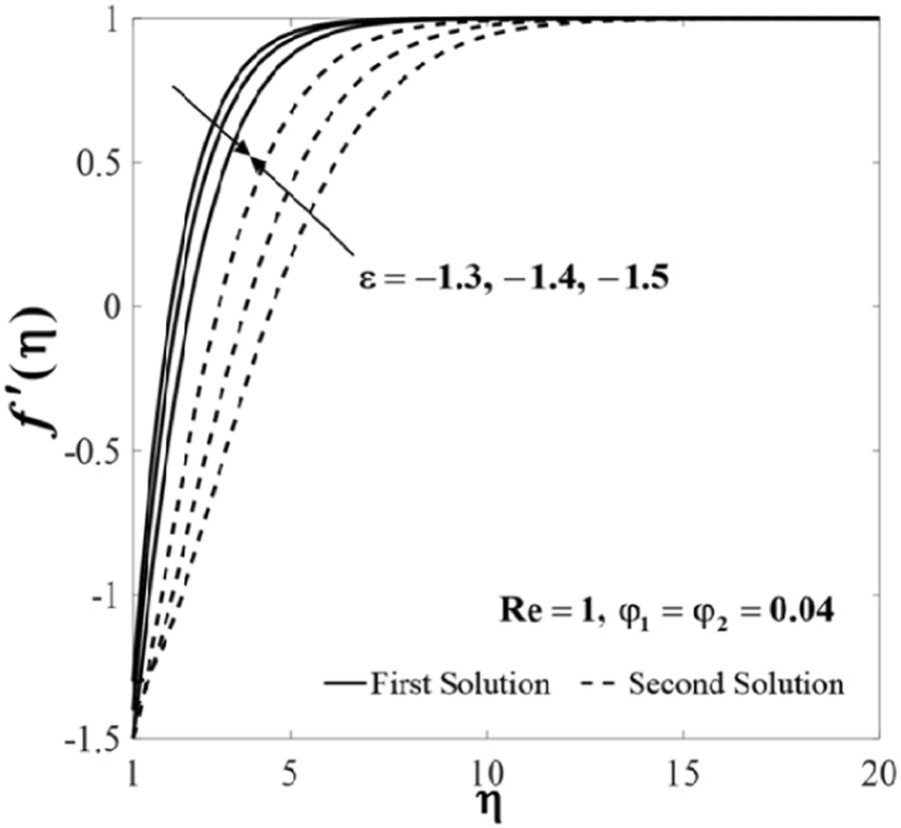
Plot of *f*′(*η*) for different values of *ε*.

**Figure 11:**
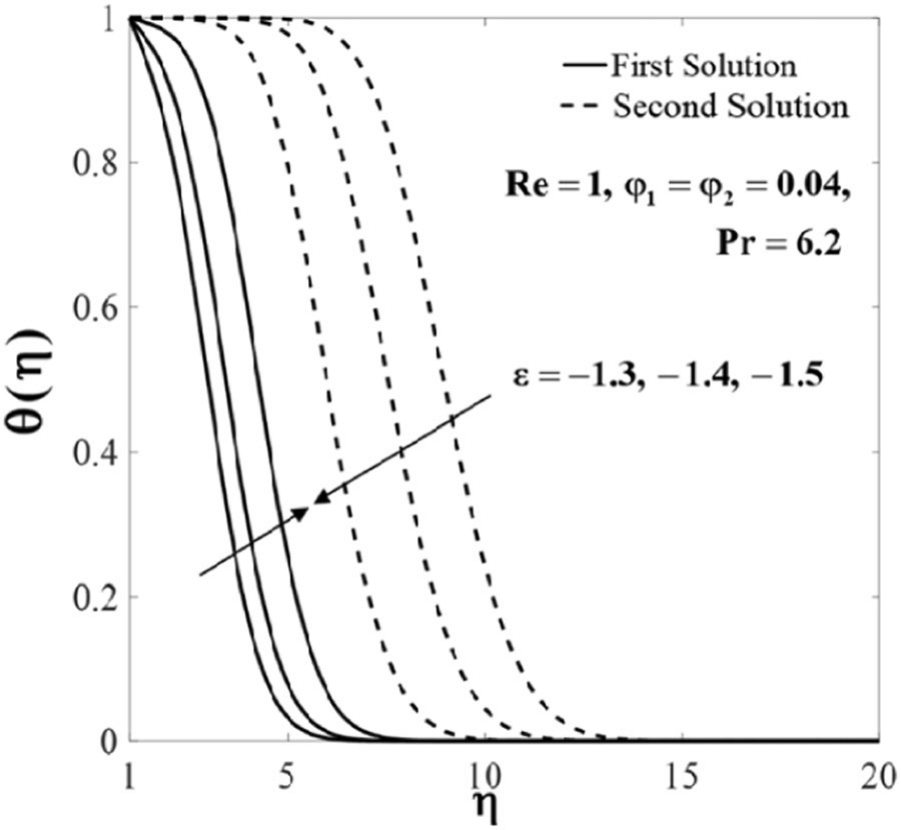
Plot of *θ*(*η*) for different values of *ε*.

Further, the velocity profiles *f*'(*η*) and the temperature profiles *θ*(*η*) for selected values of parameters are provided in Figs. 6–11. These profiles asymptotically satisfy the free stream conditions (8), and thus supports the validity of the numerical solutions. The increasing behaviour for both branch solutions of *f*'(*η*) is observed with the increase of *φ*_2_ when $\varepsilon =-1.5,{\rm{Re}}=1,{\varphi }_{1}=0.04,$ and $\Pr =6.2$, however, the observation is reversed for *θ*(*η*) as can be seen in Figs. 6 and 7, respectively. Moreover, the effect of *Re* on *f*'(*η*) and *θ*(*η*) when *ε* = −1.5, *φ*_1_ = *φ*_2_ = 0.04, and $\Pr =6.2$ are exhibited in Figs. 8 and 9. As predicted, the velocity *f*'(*η*) increases with the rise of Reynolds number *Re* and as a result, the temperature *θ*(*η*) decreases once the thermal diffusion is overcome. This observation shows similar results as those of Lok and Pop^12^ for the viscous fluid case. In addition, the effect of *ε* on *f*'(*η*) and *θ*(*η*) when $\mathrm{Re}=1,{\varphi }_{1}={\varphi }_{2}=0.04,$ and $\Pr =6.2$ are presented in Figs. 10 and 11. It can be predicted that both solutions merge at *ε* = *ε*_*c*_. This observation is consistent with the results presented in Figs. 2–5.

**Figure 12:**
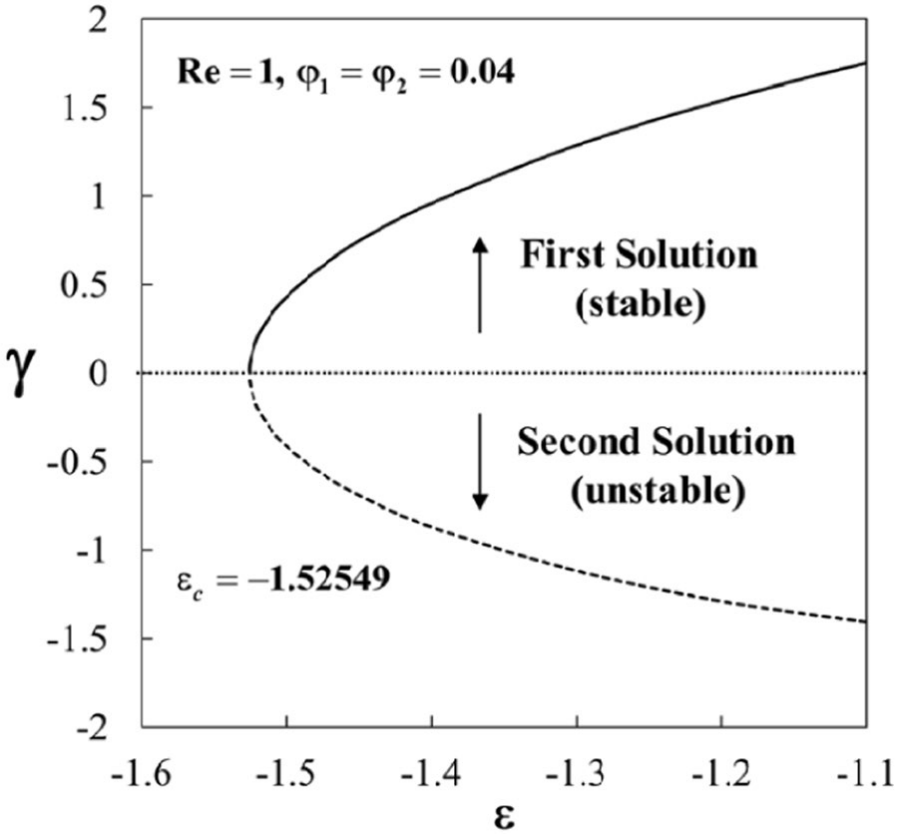
Plot of *γ* against *ε* for *F*''(1) = 1.

The variations of the smallest eigenvalues *γ* against *ε* when *φ*_1_ = *φ*_2_ = 0.04 and $\mathrm{Re}=1\,$are portrayed in Fig. 12. It is noticed that the values of *γ* are positive for the first solution, while it is negative for the second solution. Also, the values of *γ* approach to zero for both solutions when *ε* → *ε*_*c*_ = −1.52549. Thus, this finding confirms that the first solution is stable and physically reliable while the second solution is not. Besides, it is concluded that the bifurcation of the solutions happens at the critical (minimum) value *ε* = *ε*_*c*_.

Equations (6) to (8) admit dual solutions due to the reverse flow occurs in the boundary layer induced by the shrinking sheet. The occurrence of this phenomenon creates the separation of the boundary layer where the flow moves in the opposite direction as shown in Fig. 1(b). The stability of the solutions is indicated by the sign of the eigenvalue *γ*. As described in Eq. (15), there is an initial decay of disturbance when *γ* > 0 as time evolves, i.e. *e*^−*γτ*^ → 0 as *τ* → ∞. Thus, the flow is stable in the long run when *γ* > 0. In contrast, the flow is unstable when *γ* < 0 since *e*^−*γτ*^ → ∞ as  → ∞. The latter shows an increase of disturbance as time evolves. This analysis shows that the first solutions are stable and thus physically reliable in the long run, while the second solutions are not and have no physical sense. Although such solutions are deprived of physical significance, they are nevertheless of interest so far as the differential equations are concerned. These solutions are of mathematical interest since they are also solutions to the differential equations. Similar equations may arise in other situations where the corresponding solutions could have more realistic meaning^56^.

## Conclusion

In the present study, the hybrid nanofluid flow towards a stagnation point on a stretching/shrinking cylinder has been accomplished. The results were obtained through the bvp4c solver in Matlab software. The results validation was done for limiting cases where the current results were found to compare well with the existing results. The effects of the nanoparticles volume fractions (*φ*_1_ and *φ*_2_), stretching/shrinking parameter *ε*, and Reynolds number *Re* on the flow and heat transfer characteristics have been examined. From this investigation, we can draw the following conclusions:

- The findings revealed that the heat transfer rate improved in the presence of hybrid nanoparticles.
- It was found that dual solutions are possible for certain physical parameters, where the bifurcation of the solutions occurred in the shrinking region (*ε* < 0).
- The Nusselt number *Nu* enhanced with increasing values of the Reynolds number *Re*. The effect of *Re* was more dominant for the case of the stretching surface (*ε* > 0).
- The velocity increased, but the temperature decreased with the rising of *φ*_2_ and *Re*.
- Between the two solutions, only one of them is stable and physically reliable, while the other is unstable as time evolved.
